# Commentary: Bendopnea as an independent prognostic marker for adverse events in hospitalized heart failure patients: insights from a multicenter prospective cohort study

**DOI:** 10.3389/fcvm.2025.1745774

**Published:** 2026-01-20

**Authors:** Ramon Baeza-Trinidad

**Affiliations:** Internal Medicine Department, San Pedro Hospital, Logroño, Spain

**Keywords:** acute heart failure, bendopnea, diagnosis, dyspnea, heart faiIure

A commentary on Bendopnea as an independent prognostic marker for adverse events in hospitalized heart failure patients: insights from a multicenter prospective cohort study By Baeza-Trinidad R. Front. Cardiovasc. Med. (2025) 12: 1659830. doi: 10.3389/fcvm.2025.1659830

I have read with great interest the article by Wu et al. entitled “*Bendopnea as an independent prognostic marker for adverse events in hospitalized heart failure patients: insights from a multicenter prospective cohort study*” (1). The prevalence of bendopnea in decompensated heart failure patients, notably higher in those with advanced NYHA class, was similar to previously reported findings, underscoring its importance in advanced heart failure. The authors conclude that bendopnea is an independent predictor of adverse events, including mortality, in hospitalized heart failure patients. While we appreciate their reinforcement of bendopnea's prognostic value through a multicenter prospective cohort, we would like to contextualize these findings by noting that the association between bendopnea and adverse mortality-related outcomes in heart failure patients has been demonstrated previously. Nonetheless, their discussion of the symptom's diagnostic utility and potential for risk stratification is particularly valuable, especially since it represents the largest prospective study to date assessing prognosis in this population.

Since bendopnea (initially called flexo-dyspnea) was first described in the early 2010s, several studies have been conducted to evaluate the association of this symptom with poor prognosis (2, 3). Indeed, our initial study, which included 250 hospitalized patients, was the first to evaluate this fact in acute heart failure patients (4). It described a significant association with an increased mortality rate in hospitalized patients with decompensated heart failure in six months follow-up period (in univariate analysis), also reporting an increasing trend in readmission rates. This finding highlights the importance of this symptom not only as a prevalent manifestation in patients with heart failure but also as a significant prognostic factor. This symptom was also associated with other heart failure signs and symptoms, including paroxysmal nocturnal dyspnea, elevated jugular venous pressure, abdominal fullness, oliguria and orthopnea, with the latter three specifically showing a relationship with a quicker onset of dyspnea during the maneuver. This association, as well as an advanced functional class, was similar to that observed in other studies, incluiding the study by Wu et al.

Further supporting the prognostic role of bendopnea, Thibodeau et al. described an increase in heart failure admissions at 3 months and an association with cardiovascular mortality in ambulatory patients with reduced left ventricle ejection fraction, highlighting its prognostic value during stabilization (5). More recently, in a *post-hoc* analysis of the FRAGILE-HF and SONIC-HF studies, Nakade et al. reported that the presence of bendopnea at discharge was associated with higher two-year mortality (6). On the other hand, in another study conducted by our group, it was observed that the absence of bendopnea at discharge in patients who presented it on admission did not result in a decrease in mortality or readmissions after six months (7). Additionally, bendopnea has been studied in other pathologies such as aortic valve stenosis, obstructive sleep apnea syndrome, pulmonary hypertension and also in general population, though an association with prognosis in these non heart failure contexts has not been described yet (8–12).

In conclusion, it is clear that the evidence base concerning bendopnea in heart failure is robust, consistently demonstrating its relationship with a worse prognosis in short, medium, and long term follow-up. The multicenter prospective data presented by Wu et al. reinforce its association with mortality. It is appreciable that their approach, which reinforces the previously known evidence of bendopnea as a crucial and identifiable sign that should be used during clinical practice as an easily risk stratification of heart failure patients. While the prognostic value of bendopnea is well established, further investigations are needed to clarify the mechanisms underlying its changes in response to therapeutic interventions. Therefore, future research should also explore the role of bendopnea resolution as a potential biomarker for treatment strategies.
